# Bioactivity and Mycochemical Profile of Extracts from Mycelial Cultures of *Ganoderma* spp.

**DOI:** 10.3390/molecules27010275

**Published:** 2022-01-03

**Authors:** Katarzyna Sułkowska-Ziaja, Gokhan Zengin, Agnieszka Gunia-Krzyżak, Justyna Popiół, Agnieszka Szewczyk, Magdalena Jaszek, Jerzy Rogalski, Bożena Muszyńska

**Affiliations:** 1 Department of Pharmaceutical Botany, Faculty of Pharmacy, Medical College, Jagiellonian University, Medyczna 9, 30-688 Kraków, Poland; agnieszka.szewczyk@uj.edu.pl (A.S.); bozena.muszynska@uj.edu.pl (B.M.); 2 Physiology and Biochemistry Research Laboratory, Department of Biology, Science Faculty, Selcuk University, Konya 42130, Turkey; gokhanzengin@selcuk.edu.tr; 3 Department of Bioorganic Chemistry, Chair of Organic Chemistry, Faculty of Pharmacy, Jagiellonian University Medical College, 30-688 Kraków, Poland; agnieszka.gunia@uj.edu.pl; 4 Department of Pharmaceutical Biochemistry, Faculty of Pharmacy, Jagiellonian University Medical College, 30-688 Kraków, Poland; justyna.popiol@uj.edu.pl; 5 Department of Biochemistry and Biotechnology, Maria Curie-Skłodowska University, Akademicka 19, 20-033 Lublin, Poland; magdalena.jaszek@poczta.umcs.lublin.pl (M.J.); jerzy.rogalski@mail.umcs.pl (J.R.)

**Keywords:** antioxidant activity, cholinesterase inhibition, cytotoxic activity, *Ganoderma* spp., indole compounds, mycelial cultures, phenolic compounds, tyrosinase inhibition, α-amylase inhibition

## Abstract

Fungal mycelium cultures are an alternative to natural sources in order to obtain valuable research materials. They also enable constant control and adaptation of the process, thereby leading to increased biomass growth and accumulation of bioactive metabolites. The present study aims to assess the biosynthetic potential of mycelial cultures of six *Ganoderma* species: *G. adspersum*, *G.* *applanatum*, *G. carnosum*, *G. lucidum*, *G. pfeifferi*, and *G. resinaceum*. The presence of phenolic acids, amino acids, indole compounds, sterols, and kojic acid in biomass extracts was determined by HPLC. The antioxidant and cytotoxic activities of the extracts and their effects on the inhibition of selected enzymes (tyrosinase and acetylcholinesterase) were also evaluated. The total content of phenolic acids in the extracts ranged from 5.8 (*G. carnosum*) to 114.07 mg/100 g dry weight (d.w.) (*G. pfeifferi*). The total content of indole compounds in the extracts ranged from 3.03 (*G. carnosum*) to 11.56 mg/100 g d.w. (*G. lucidum*) and that of ergosterol ranged from 28.15 (*G. applanatum*) to 74.78 mg/100 g d.w. (*G. adspersum*). Kojic acid was found in the extracts of *G. applanatum* and *G. lucidum*. The tested extracts showed significant antioxidant activity. The results suggest that the analyzed mycelial cultures are promising candidates for the development of new dietary supplements or pharmaceutical preparations.

## 1. Introduction

The genus *Ganoderma* was described in 1881 and initially comprised only one species—*G. lucidum* (Curtis) Karst. [1]. Species in the genus *Ganoderma* are considered to be important natural sources of compounds with medicinal properties. Their therapeutic effects have been known for thousands of years. They are most popular in Asia, especially in the field of Traditional Chinese Medicine (TCM). These species are also used traditionally in some West African countries, such as Nigeria, for treating skin diseases, hypertension, and digestive disorders [2,3]. Scientific research has confirmed the broad-spectrum effects of isolated compounds and extracts from *Ganoderma* spp., e.g., antitumor, immunomodulatory, antioxidant, anti-inflammatory, antiallergic, neuroprotective, hepatoprotective, hypoglycemic, hypotensive, antimicrobial, antiviral, and antimalarial effect [4,5]. The most important groups of compounds are triterpenes (GTs-*Ganoderma* triterpenes) and polysaccharides [6]. Because of their chemical constituents, the extracts of *Ganoderma* spp. are an interesting object of biotechnological research. Previous studies have mainly focused on mycelial cultures of *G. lucidum*—a species with the broadest spectrum of activity [7]. Numerous studies were conducted on polysaccharides and ganoderic acids obtained from mycelial cultures of *G. lucidum* in liquid media in flasks or bioreactors [8]. The obtained scientific data indicate that proper selection of conditions, such as temperature, pH, and nutrient composition, plays an important role in biomass production and accumulation of metabolites [9]. Many processes used by mycelial cultures of higher fungi to synthesize biologically active compounds or to break down environmental pollutants have already been patented [10]. The possibility of using such processes on a larger scale depends mainly on the development of suitable culture conditions and the transfer of the process from laboratory scale to industrial scale. Continuous advancements in biotechnology and gaining in-depth knowledge on the properties of mushrooms have enabled us to design new biotechnological processes.

Mycelial cultures have enormous potential to produce bioactive compounds; however, this potential remains to be fully understood. Hence, research focused on the identification of metabolites isolated from mycelial cultures offers the possibility of developing new substances with potential applications in medicine discovery [11].

The present study aimed to evaluate the bioactivity of extracts from mycelial cultures, which are considered as sources of compounds with health promoting properties. The study covered mycelial cultures of six *Ganoderma* species: *G*. *adspersum*, *G*. *applanatum*, *G*. *carnosum*, *G*. *lucidum*, *G*. *pfeifferi,* and *G*. *resinaceum*. The contents of phenolic acids, indole compounds, amino acids, sterols, and kojic acid were determined in extracts of culture biomass by using the reversed-phase high-performance liquid chromatography method with diode array detection (DAD) and UV detection. Total phenol content (TPC) and total flavonoid content (TFC) were determined by spectral methods. The antioxidant potential of the extracts was assessed by various in vitro methods: DPPH (1,1-diphenyl-2-picrylhydrazyl) test, ABTS ((2,2′-azinobis-(3-ethylbenzthiazolin-6-sulfonic acid), FRAP (Ferric Reducing Ability of Plasma), CUPRAC (CUPric Reducing Antioxidant Capacity), MCA (metal chelating activity), and PHD (phosphomolybdenum assay). Inhibition of the enzymes tyrosinase, acetylcholinesterase, and butyrylcholinesterase was also determined. The obtained extracts were tested for cytotoxic properties against B16F10 mouse melanoma cells. The ability of the study extracts to inhibit melanogenesis in B16F10 cells was also assessed. This is the first report to provide evidence of potential therapeutic value of biomass extracts from the studied mycelial cultures.

## 2. Results and Discussion

### 2.1. Mycelial Cultures

*Ganoderma* species, namely *G. adspersum, G. applanatum, G. carnosum, G. lucidum, G. pfeifferi*, and *G. resinaceum*, are known to be used in traditional medicine for thousands of years. They were typically used as raw materials in traditional medicine in Asia or West Africa. These species also started to appear in conventional medicine applications in Asia over time. Presently, there is a growing interest in these mushroom raw materials even in other parts of the world. Currently, research on natural raw materials is increasingly involving the use of biotechnology methods. Mycelial cultures are now a current object of biotechnological research. This method allows significant shortening of the time required to obtain research materials and optimization of physicochemical conditions. Additionally, mycelial cultures ensure repeatability of cultivation conditions, leading to the generation of biomass samples with a specific and predictable composition. Mycelial cultures also enable us to obtain biomass enriched with specific components, e.g., through appropriate precursors of metabolic pathways or elicitors. 

The possibility of maintaining mycelial cultures has made them a suitable alternative to materials obtained from natural habitats. In the experimental cultures, *Ganoderma* mycelium grew in the form of spherical, compact aggregates of a bright cream color. For all the culture series conducted in a liquid medium, the mean values of the biomass growth parameter were determined as dry weight (d.w.) obtained from 1 L of the medium. The highest mean increase in biomass was observed for *G. pfeifferi* (6.67 g/L), while the lowest was noted for *G. adspersum* (2.04 g/L). For the other species, the mean increase in biomass was as follows: *G. applanatum*—4.06 g/L, *G. carnosum*—2.88 g/L, *G. lucidum*—5.12 g/L, and *G. resinaceum*—5.02 g/L. The dynamics of mycelium growth in the same medium did not differ from those reported in our earlier studies [12,13].

### 2.2. Mycochemical Analyses

The quantitative and qualitative analyses of the selected organic compounds demonstrated an interspecies variability. The results of mycochemical analyses are shown in Table 1.

Phenolic acids are compounds commonly found in fungal species. Phenolic acids possess broad-spectrum bioactivity, including antioxidant, hypolipemic, immunostimulant, antitumor, anticoagulant, anti–inflammatory, antispasmodic, and choleretic properties [14]. The content of specific phenolic acids in the studied material varied, depending on the species. In the studied extracts, five phenolic acids were determined: gallic acid, protocatechuic acid, 3,4-dihydroxyphenylacetic acid, *p*-hydroxybenzoic acid, and caffeic acid. Protocatechuic acid was present in all tested extracts from biomass obtained from mycelial cultures, and its highest content was determined in *G. carnosum*—3.95 mg/100 g dry weight (d.w.). The highest gallic acid content was found in the extract of *G. pfeifferi*—34.31 mg/100 g d.w. The highest content of 3,4-dihydroxyphenylacetic acid was found in the extract of *G. pfeifferi*—77.37 mg/100 g d.w., while *p*-hydroxybenzoic was present in the extract of *G. resinaceum* in the amount of 2.17 mg/100 g d.w. The most significant amount of caffeic acid was detected in the extracts of *G. applanatum*—3.36 mg/100 g d.w. *G. applanatum* was the only species in which all the five abovementioned acids were identified. The highest TPC was determined in *G. pfeifferi* biomass extract—114.07 mg/100 g d.w.), while the lowest TPC was found in the extract of *G. resinaceum*—44.71 mg/100 g d.w.). Gallic acid possesses antioxidant, bacteriostatic, and anticancer properties. Additionally, it shows an antiseptic and astringent effect, and antiperspirant properties [15]. Protocatechuic acid has proven antifungal, anti-inflammatory, antioxidant, chemopreventive, and hepatoprotective activities [15]. Caffeic acid possesses antioxidant, chemopreventive, anticancer, choleretic, and antibacterial properties. *p*-Hydroxybenzoic acid also demonstrates antimicrobial properties [15]. The content of phenolic acids in *Ganoderma* species has also been investigated by other studies. The content of phenolic compounds in the methanolic extract of *G. applanatum* was 9.98 ± 0.01 mg/g d.w. (chlorogenic acid equivalent). A quantitative analysis revealed the following phenolic acid content: protocatechuic acid (18 µg/g), gallic acid (20 µg/g), caffeic acid (44 µg/g), and coumaric acid (24 µg/g) [16]. In a methanolic extract of *G. lucidum*, the content of phenolic acids was 9.35 ± 0.03 mg/g d.w. (chlorogenic acid equivalent). Among the phenolic compounds, gallic acid was dominant—1103 µg/g [16]. Analyses conducted by Rašeta et al. [17] confirmed the occurrence of the following phenolic acids in ethanolic extracts from *G. applanatum*, *G. lucidum*, *G. pfeifferi*, and *G. resinaceum* fruiting bodies: hydroxybenzoic acid, protocatechuic acid, *p*-coumaric acid, and caffeic acid. Chlorogenic acid was identified in *G. pfeifferi* fruiting bodies. Vanillic acid was the quantitatively dominant phenolic acid in the ethanolic extracts of *G. applanatum*, *G. lucidum*, and *G. pfeifferi* fruiting bodies in the amount of 12.1, 7.1, and 7.6 µg/g d.w., respectively [17]. Additionally, the total phenolics and total flavonoids in the tested extracts were determined. The TPC ranged from 10.57 mg gallic acid equivalent (GAE)/g for *G. adspersum* to 16.56 mg GAE/g for *G. carnosum*. The total content of flavonoids ranged from 0.21 mg rutin equivalent (RE)/g in *G. adspersum* and *G. pfeifferi* to 0.57 mg RE/g in *G. resinaceum* (Table 2).

The estimated total content of these compounds correlated with the antioxidant activity of the tested extracts. The content of indole compounds in the mycelial cultures of *Ganoderma* has not yet been comprehensively evaluated. Qualitative analysis of non-hallucinogenic indole derivatives confirmed the presence of L-tryptophan in the extracts of all tested species. The content of L-tryptophan ranged from 10.58 mg/100 g d.w. in *G. lucidum* to 3.03 mg/100 g d.w. in *G. carnosum*. In the extracts of *G. applanatum*, *G. lucidum*, and *G. pfeifferi*, the melatonin content was 0.02, 0.98, and 0.012, respectively (Table 1). Melatonin (*N*-acetyl-5-methoxytryptamine) is one of the non-hallucinogenic indole compounds, the presence of which has been confirmed in some edible Basidiomycota species. Its precursor is tryptophan, which is transformed into 5-hydroxytryptophan, 5-hydroxytryptamine (serotonin), N-acetylserotonin, and finally into 5-methoxy-*N*-acetyltryptamine, i.e., melatonin, by enzyme activity. This compound is involved in the regulation of sleep, mood, and reproduction. It coordinates the work of the biological clock that controls the circadian rhythm, has antiaging and anticancer properties, and regenerates the processes of cell renewal. Melatonin is also a powerful antioxidant, and it neutralizes free radicals, e.g., hydroxyl (• OH). Thus, it can reduce the damage caused by some types of Parkinson’s disease. Some studies have also reported its effectiveness in treating Alzheimer’s disease [18]. Melatonin also plays a neuroprotective role in neurodegenerative diseases. It also has immunomodulatory, anti-inflammatory, cardioprotective, blood pressure lowering, and lipid and glucose metabolism regulation properties. Indole compounds support the proper functioning of the nervous system by acting as neurotransmitters and their precursors (tryptophan and 5-hydroxytryptophan) [18]. Indole derivatives play an important role in maintaining the homeostasis of organisms. They act as functional neurotransmitters and their precursors and influence the functioning of the nervous system. In higher fungi, these compounds are represented by tryptophan and tryptamine derivatives [19]. Our previous study proved their occurrence in the fruiting bodies of *Phellinus* spp. [20] and in the mycelial cultures of *Fomitopsis betulina* [12]. In the analyzed biomass, the presence of ergosterol and trace amounts of ergosterol peroxide was detected (Table 1). Sterols occur naturally in free or conjugated form. Higher fungi produce 5,7,22-ergostatrien-3β-ol (ergosterol) as the primary sterol with two double bonds in the sterol ring structure instead of the single bond characteristic in plants. Ergosterol is becoming an important research topic, as its content is mostly associated with the different phases of mushroom growth. After photolysis, this compound can be converted to vitamin D_2_. Ergosterol also has antioxidant and anti-inflammatory properties. Trace amounts of ergosterol peroxide were found qualitatively in all the tested samples. Ergosterol peroxide (5α, 8α-epidioxy-22 E-ergosta-6,22-dien-3β-ol), like ergosterol, is present in most species of Basidiomycota. This compound has broad-spectrum therapeutic effects, including antimicrobial, cytotoxic, and immunosuppressive effects. Ergosterol peroxide is a promising drug development candidate and contributes to the health-promoting effects of medicinal mushrooms [21]. In the *n*-hexane extract from fruiting bodies of *G. applanatum*, the following ergosterol derivatives were identified: ergosta-7,22-dien-3β-one, ergosta-7,22-dien-3β-ol, and three stearyl esters of linoleic acid: 3β-linoleyloxyergosta-7,22–diene, 3β-linoleyloxyergost-7-ene, and 3β-linoleoloxyergosta-7,24 (28)-diene. Presumably, these free sterols play a role in the growth of the fruiting body [22]. In a study comparing the content of sterols and fatty acids of two species—*G. lucidum* and *G. sinense*, the content of ergosterol—a component of the fungal cell membrane—was determined [23].

In traditional medicine, extracts from raw materials of natural origin are used rather than individual compounds. There is evidence that crude extracts often exhibit more significant biological activity in vitro and/or in vivo than isolated components at an equivalent dose. Pure drugs that are industrially produced or isolated from plants or mushrooms may be attractive because of their high activity against human diseases, but they have disadvantages. They rarely have the same degree of activity as the crude extract at comparable concentrations or doses of the active ingredient. In traditional medicine, many years of effective use of extracts made of natural raw materials, including mushroom raw materials, make it necessary to find a pharmacological and therapeutic justification for the superiority of many of them as compared to individual ingredients. The synergistic effects can be verified through detailed pharmacological studies and controlled clinical trials as compared to synthetic reference drugs. This enables their use in treating diseases that have been treated only with synthetic drugs.

### 2.3. Antioxidant Activity

Mushrooms are important cornerstones in the search for natural antioxidants by scientists. In this context, studies on the antioxidant properties of mushrooms have gained in importance in recent years [24,25]. In the current study, the antioxidant properties of six *Ganoderma* species were determined by various chemical assays, and the results are shown in Table 2. Two radicals, namely DPPH and ABTS, were used to assess the radical scavenger potential of the tested extracts. *G. resinaceum* (DPPH: 6.74 mg TE/g and ABTS: 11.60 mg TE/g) demonstrated the best activity in both radical scavenging assays, followed by *G. applanatum* (DPPH: 5.83 mg TE/g and ABTS: 11.29 mg TE/g) and *G. adspersum* (DPPH: 5.61 mg TE/g and ABTS: 10.16 mg TE/g). Similar to the radical scavenging results, *G. resinaceum* (FRAP: 10.16 mg TE/g and CUPRAC: 31.78 mg TE/g) had the strongest reducing properties, reflecting an electron donating ability.

*G. pfeifferi* extract was the second highest in terms of ability in the CUPRAC assay. In the PHD assay, the samples were rated in the following order: *G. resinaceum* > *G. lucidum* > *G. carnosum* > *G. applanatum* > *G. pfeifferi* > *G. adspersum*. The order was important because both phenolic and nonphenolic antioxidants can play a role in the PHD assay, which is known as the total antioxidant assay. The chelation of transition metals is considered to be one of the important mechanisms that inhibit the production of hydroxyl radicals by the Fenton and Haber–Weiss reaction. All the extracts demonstrated iron (II) chelation, and the best was *G. applanatum* with 8.98 mg EDTAE/g. *G. pfeifferi*, however, demonstrated the lowest metal chelating ability. Several studies have been conducted on the antioxidant properties of various *Ganoderma* species. For example, Zengin et al. [26] investigated the chemical profile and biological abilities of two *Ganoderma* species (*G. applanatum* and *G. resinaceum*) from Turkey. The extracts showed stronger antioxidant abilities than those reported by our studies. For example, the DPPH radical scavenging ability ranged from 17.01 to 59.24 mg TE/g in the tested Turkish *Ganoderma* species, which is higher than those of the six *Ganoderma* species (5.01–6.74 mg TE/g) tested in our study. Similarly, different results for *Ganoderma* species have been reported by several researchers [27,28,29]. The different mycochemical compositions could explain the differences in the obtained results. In this sense, as shown in the present study, varying levels of different phenolic components were found in the individual *Ganoderma* species [30,31,32]. As a further approach, these differences could be explained by the use of different culture media for fungal growth [32,33,34].

### 2.4. Inhibition of Cholinesterase and Amylase

Cholinesterase is a pharmaceutical target in the treatment of Alzheimer’s disease. This is because its inhibitory activity increases the level of acetylcholine in the synaptic cleavage. The increased acetylcholine levels could help to improve cognitive functions in patients with Alzheimer’s disease. Keeping this in mind, some chemicals have been produced to alleviate the observed symptoms in patients with Alzheimer’s disease, but in the long term, most of them have unpleasant side effects, such as gastrointestinal disturbances or toxicity. Hence, we need to find safe and effective cholinesterase inhibitors from natural sources. In the present study, the cholinesterase inhibitory effects of the tested *Ganoderma* species are shown in Table 3.

Three extracts displayed activity against acetylcholinesterase (AChE), and *G. adspersum* extract demonstrated a significant inhibitory effect (1.22 mg GALAE/g). In recent studies, butrylcholinesterase (BChE) is considered as an important enzyme for treating Alzheimer’s disease. Regarding the inhibition of BChE by the tested *Ganoderma* species, all extracts inhibited BChE (0.60–1.13 mg GALAE). The best BChE inhibitory effect was observed for *G. applanatum* extract, while *G. pfeifferi* extract demonstrated the lowest inhibitory effect. A literature survey demonstrates that several studies have reported important cholinesterase inhibitory effects by some *Ganoderma* species. For example, Zengin et al. [26] reported that the water and methanolic extracts of two *Ganoderma* species (*G. applanatum* and *G. resinaceum*) inhibited AChE (0.62–1.47 mg GALAE/g) and BChE (0.70–2.94 mg GALAE/g). Some authors have also isolated some anticholinesterase agents from specimens of *Ganoderma* [35,36]. Altogether, the members of the genus *Ganoderma* could be valuable candidates as sources of natural cholinesterase inhibitors for the development of anti-Alzheimer’s disease drugs. Diabetes mellitus is one of the major health problems in the world, and its prevalence is increasing each year. Researchers are therefore looking for effective treatment strategies to control this problem. Keeping this in mind, carbohydrate-hydrolyzing enzymes are the main targets for controlling blood glucose level in patients with diabetes. Amylase hydrolyzes the α-(1,4)-glycosidic bond in starch, leading to increased blood glucose levels. From this perspective, inhibiting the amylase enzyme could control the blood glucose level in patients with diabetes following a high carbohydrate-rich diet. Several oral antidiabetic drugs (acarbose and voglibose, etc.) have been produced for this purpose, but they have several side effects, such as diarrhea, constipation, nausea, and vomiting. Therefore, natural enzyme inhibitors are needed to manage blood glucose levels in patients with diabetes. For this purpose, the amylase inhibitory effects of the six *Ganoderma* species were investigated in the present study. All the tested *Ganoderma* extracts exhibited close amylase inhibitory effects, and the best amylase inhibitory effect was found in *G. adspersum* extract (0.15 mmol ACAE/g). The weakest amylase inhibitory effect was shown by *G. resinaceum* (0.13 mmol ACAE/g). In an earlier study by Deveci et al. [37], the amylase inhibitory effect of the hexane extract of *G. adspersum* at the concentration of 1 mg/mL was found to be 96.96%. In the same study, however, the methanolic extract at the same concentration demonstrated an inhibitory effect of 14.40%. In another study by Yalcin et al. [38], the amylase inhibitory effects of the methanolic extracts of *G. carnosum* and *G. pfeifferi* were found to be 352.33 and 211.83 mg ACE/g, respectively. Chen et al. [39] investigated the amylase and glucosidase inhibitory effects of five *Ganoderma* species and their triterpenoids, and *G. lucidum* triterpenes demonstrated the strongest inhibitory effect with the lowest IC_50_ values. Similar results of isolated compounds from the members of *Ganoderma* were also reported by several authors [40,41,42].

Although the extracts of the tested *Ganoderma* species demonstrates good results for cholinesterase and amylase inhibitory activities, these results are insufficient for their direct use in patients with Alzheimer’s disease and diabetes mellitus. Therefore, we emphasize that further research, including animal and toxicity tests, is needed to support their clinical application. The present results could provide hope to scale up the application from the laboratory bench to clinical settings.

### 2.5. Tyrosinase Inhibition Assay and Tests in Mouse Melanoma B16F10 Cells

**Figure 1:**
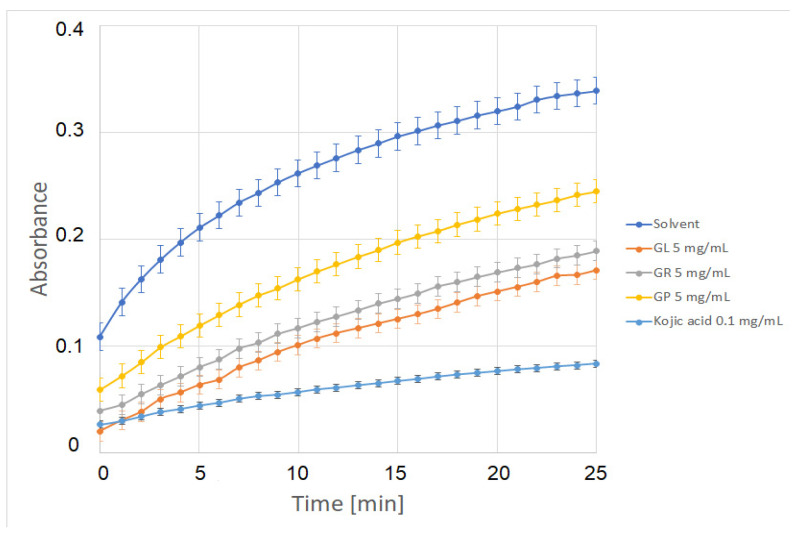
Changes in absorbance at 475 nm following 25 min incubation of buffer with tyrosinase and 0.5 mM L–DOPA with or without the tested extracts of *G. lucidum* (GL), *G. resinaceum* (GR), and *G. pfeifferi* (GP) at the concentration of 5 mg/mL as well as 0.1 mg/mL kojic acid.

Incubation of the reaction mixture containing mushroom tyrosinase in phosphate buffer and L–DOPA as a substrate leads to the formation of dopachrome, which can be identified as a red–brown colored compound. The addition of an inhibitor causes the color to fade (become less intense or even undetectable). The amount of dopachrome formed is measured in terms of absorption at a particular wavelength. Three extracts tested in the present study (*G. lucidum*, *G. pfeifferi*, and *G. resinaceum*) demonstrated moderate tyrosinase inhibitory properties. These extracts prevented dopachrome formation, which decreased the absorbance value at 475 nm, as shown in Figure 1.

All the extracts were weaker inhibitors when compared with kojic acid at the concentration of 0.01 mg/mL (Table 4).

The strongest inhibition of tyrosinase was observed for *G. lucidum* extract, which inhibited the activity of the enzyme by 50.53% at 5 mg/mL concentration. Effective tyrosinase inhibitors, such as hydroquinone or arbutin, however, have undesirable effects. For instance, hydroquinone exhibits cytotoxic effects on melanocytes and is suspected of exerting a mutagenic effect on mammalian cells [43]. Mushrooms can synthesize numerous compounds that are capable of inhibiting tyrosinase. These include kojic acid, azelaic acid, or 3,4-dihydroxybenzaldehyde. In the present study, the capacity of kojic acid accumulation was analyzed in the mycelial cultures at the level of 0.14 and 0.39 mg/100 g d.w. in *G. applanatum* and *G. lucidum*, respectively (Table 1). Kojic acid is the most thoroughly studied inhibitor of the tyrosinase enzyme and is obtained from *Aspergillus* growing on corn kernels. Because of its good solubility in water, it is widely used in cosmetic preparations. In addition to its depigmenting properties, kojic acid also has an antibacterial effect and prevents the formation of free radicals. Cosmetic preparations with this component not only lighten discoloration, but they also have an antiwrinkle and moisturizing effect. In addition, kojic acid inhibits the uptake of oxygen, which is essential for enzymatic browning [44].

Extracts were tested at a 0.15 mg/mL concentration in the MTT viability assay to assess their cytotoxic effect on B16F10 mouse melanoma cells. Only noncytotoxic concentrations were used in assays for antimelanogenic activity. Therefore, kojic acid (0.03 mg/mL) was also used in the assay as a reference depigmenting agent. The tested extracts at the concentration of 0.15 mg/mL displayed varying cytotoxicity levels, ranging from 43.29% ± 1.25% for the most cytotoxic extract of *G. carnosum* to 93.89% ± 2.72% for the least cytotoxic extract of *G. lucidum* (data not shown).

Based on the results of the tyrosinase inhibition assay and the MTT viability assay in B16F10 cells, *G. lucidum*, *G. pfeifferi*, and *G. resinaceum* (0.15 mg/mL) were chosen for the assessment of melanogenesis inhibition properties in B16F10 mouse melanoma cells. Kojic acid (0.03 mg/mL) was used as a reference compound. In the performed study, only kojic acid showed an ability to inhibit the αMSH-induced melanin production, while the tested extracts did not demonstrate such an activity. Despite encouraging results for antityrosinase properties, the B16F10 cell assay did not confirm the antimelanogenic effect of the tested species. These findings might be due to the low concentration of extracts used, as higher concentrations are cytotoxic to B16F10 cells (data not shown).

## 3. Materials and Methods

### 3.1. Origin of Mycelial Cultures

The study materials consisted of mycelial cultures of 6 selected *Ganoderma* species: *G. adspersum, G. applanatum, G. carnosum, G. lucidum, G. pfeifferi*, and *G. resinaceum.* Mycelial cultures were obtained from the Department of Biochemistry and Biotechnology, Institute of Biological Sciences, Faculty of Biology and Biotechnology, Maria Curie-Skłodowska University in Lublin.

### 3.2. Initial Mycelial Cultures

The initial cultures were grown on a solid Oddoux medium with own modifications (in a range of macro and microelement concentrations) [45]. The medium was sterilized for 20 min at 121 °C and 0.1 MPa (Asve, Warsaw, Poland). The cultures were grown on 60 mm Petri dishes at 22 ± 2 °C and subcultured every 3 weeks.

### 3.3. Experimental Mycelial Cultures

Experimental mycelial cultures were initiated by transferring a fragment of the biomass from an agar medium to an Erlenmeyer flask (300 mL) containing 100 mL of liquid Oddoux medium. The cultures were maintained under the same conditions as the initial cultures by using a rotary shaker (Altel, Łódź, Poland) at 140 rpm with an amplitude of 35 mm for 3 weeks.

### 3.4. Extraction of Biomass

For preparing methanolic extracts, 4 g samples of lyophilized (Labconco, Kansas City, MO, USA) and pulverized material (biomass of each species) were weighed. The material was then extracted with 50 mL methanol (STANLAB, Lublin, Poland) by sonication in an ultrasonic bath (POLSONIC 2, Warsaw, Poland) for 30 min. Next, the extracts were centrifuged (MPW-342, Med. Instruments, Warsaw, Poland) for 10 min at 4300 rpm. Methanol in the supernatant was allowed to evaporate at 22 ± 2 °C. After evaporation, the dry residue was dissolved quantitatively in HPLC-grade methanol. Before the HPLC analysis, the obtained extracts were filtered through sterilized syringe filters (0.22 μm, Millex^®^GP, Millipore).

### 3.5. Mycochemical Analysis

The chemical analysis was performed with the HPLC-DAD method, as described previously [12,46]. Methanolic extracts obtained in the process were used for these estimations. An HPLC-DAD system (Merck-Hitachi, Merck KGaA, Darmstadt, Germany) and a Purospher RP-18e analytical column (4 × 250 nm, 5 mL; Merck) were used. UV spectra were recorded in the 220–350 nm range. The peaks were identified by comparing UV spectra and retention times with those of standard compounds. The qualitative analyses were supplemented with the standard internal method. Chemical compounds were quantified with the calibration curve method against their standards. Chemical standards of phenolic acids, indole derivatives, and sterols were purchased from Sigma–Aldrich (Darmstadt, Germany) and Fluka Chemie GmbH (Buchs, Switzerland). Solvents for HPLC analysis were purchased from Merck, Darmstadt, Germany.

### 3.6. TPC Evaluation

The TPC was determined using a modified Folin-Ciocâlteu method [47] with a 96-well plate (Thermo–Multiscan, Thermo Scientific, Vantaa, Finland). Each sample (50 μL) was mixed with diluted Folin-Ciocalteu reagent (Sigma–Aldrich, Darmstadt, Germany) (100 μL, 1:9, *v*/*v*) and then mixed with sodium carbonate (2%, 75 μL). The mixture was incubated in dark for 2 h at 22 ± 2 °C. The absorbance values were then read at 765 nm. Gallic acid was used as a standard, and the results are expressed as a gallic acid equivalent (mg GAE/g).

### 3.7. TFC Evaluation

The TFC was determined using the method reported by Zengin and Aktumsek [47]. Briefly, 200 μL sample was mixed with AlCl_3_ (2% in methanol). The mixture was then incubated for 15 min at 22 ± 2 °C. The absorbances were read at 415 nm. Rutin (Sigma–Aldrich, Darmstadt, Germany) was used as a standard flavonoid, and the results are expressed as equivalent of rutin (mg RE/g).

### 3.8. DPPH Radical Scavenging Assay

The sample (50 μL) was mixed with methanolic DPPH solution (0.004%), and the mixture was then incubated for 30 min at 22 ± 2 °C. The absorbance values were read at 517 nm. Trolox was used as a standard antioxidant, and the results are expressed as equivalent of Trolox (mg TE/g) [47].

### 3.9. ABTS Assay

The radical scavenging activity of samples toward the ABTS radical cation was evaluated according to the method reported by Zengin and Aktumsek [47]. The prepared ABTS (Sigma–Aldrich, Darmstadt, Germany) radical solution was used after 12 h of incubation at 22 ± 2 °C. The sample (25 μL) was mixed with the ABTS radical solution (200 μL) and after incubation for 30 min at 22 ± 2 °C, the absorbance values were recorded at 734 nm. Trolox was used as a standard antioxidant, and the results are expressed as the equivalent of Trolox (mg TE/g).

### 3.10. FRAP Assay

The sample (25 μL) was mixed with the FRAP (Sigma–Aldrich, Darmstadt, Germany) solution (200 μL), and the mixture was incubated for 30 min at 22 ± 2 °C. The absorbance values were then read at 595 nm. Trolox was used as a standard antioxidant, and the results are expressed as the equivalent of Trolox (mg TE/g) [47].

### 3.11. CUPRAC Assay

Sample solution (25 μL) was added to a premixed reaction mixture (200 μL) containing CuCl_2_ (1 mL, 10 mM), neocuproine (1 mL, 7.5 mM), and NH_4_Ac buffer (1 mL, 1 M, pH 7.0). The absorbance values of the sample and blank were read at 450 nm after a 30-min incubation at 22 ± 2 °C. Trolox was used as a standard antioxidant, and the results are expressed as the equivalent of Trolox (mg TE/g) [16].

### 3.12. PHD Assay

The sample solution (100 μL) was mixed with 2 mL of reagent solution. The reagent solution contained 0.6 M sulfuric acid, 28 mM sodium phosphate, and 4 mM ammonium molybdate. After 90 min incubation, the absorbance of the sample was read at 695 nm. Trolox was used as a standard antioxidant, and the results are expressed as the equivalent of Trolox (mmol TE/g) [48].

### 3.13. MCA Assay

The sample solution (100 μL) was mixed with an FeCl_2_ solution (50 mL, 2 mM). Next, 5 mM of ferrozine (100 μL) was added to initiate the reaction. The reaction mixture was incubated for 10 min at 22 ± 2 °C, and the absorbance values were read at 562 nm. EDTA (Sigma–Aldrich, Darmstadt, Germany) was used as a standard chelator, and the results are expressed as the equivalent of EDTA (mg EDTAE/g) [48].

### 3.14. α-Amylase Inhibitory Activity

The sample solution was mixed with an α-amylase solution (ex-porcine pancreas, EC 3.2.1.1, Sigma–Aldrich, Darmstadt, Germany) (50 μL) in phosphate buffer (pH 6.9 with 6 mM sodium chloride) in a 96-well microplate and incubated for 10 min at 37 °C. The reaction was then initiated with the addition of starch solution (50 μL, 0.05%). The mixture was incubated for 10 min at 37 °C. HCl (25 μL, 1 M) was added to stop the reaction, and an iodine-potassium iodide solution (100 μL) was added. The absorbance values were read at 630 nm. Acarbose was used as a positive control, and the results are expressed as the equivalent of acarbose (mmol ACE/g sample) [48].

### 3.15. Cholinesterase Inhibitory Activity

Briefly, 100 µL sample solution was mixed with DTNB ((5,5-dithio-bis(2-nitrobenzoic) acid, 125 µL). An enzyme solution (AChE or BChE) was then added, and the mixture was incubated for 15 min at 22 ± 2 °C. Subsequently, the substrate (ATCI or BTCl) was added. After 10 min incubation, the absorbance values were read at 405 nm. Galantamine (Sigma–Aldrich, Darmstadt, Germany) was used as a positive control, and the results are expressed as the equivalent of galantamine (mg GALAE/g sample) [49].

### 3.16. Tyrosinase Inhibition Assay

Mushroom tyrosinase and L-DOPA were purchased from Sigma–Aldrich (Darmstadt, Germany). Tyrosinase inhibition activity was determined using the method described by Saghaie et al. [50] with a slight modification. Briefly, the tested extracts were dissolved in 33.3% dimethyl sulfoxide (DMSO) solution (Sigma–Aldrich, Darmstadt, Germany). Experiments were performed in 96-well plates in triplicates. Phosphate buffer (170 µL, 50 mM, pH 6.5) containing mushroom tyrosinase (final concentration of 1 U/mL) and 10 µL of the tested extract were mixed in a single well (final concentration of 5 mg/mL), and the plate was incubated at room temperature for 5 min. Next, 20 µL of L-dihydroxyphenylalanine (L-DOPA, final concentration of 0.5 mM) was added, and the plate was further incubated at room temperature for 25 min. DMSO solution (10 µL) was used instead of the tested extracts as a negative control. Kojic acid (Alfa Aesar, Kandel, Germany) dissolved in DMSO solution was used as a reference tyrosinase inhibitor (final concentration of 0.1 mg/mL). The amount of dopachrome produced in the reaction was measured at 475 nm using a microplate reader (Spectra iD3 Max, Molecular Devices, San Jose, CA, USA), yielding the following results: AC—absorbance of the negative control, AT—absorbance with the tested extract. The percentage of tyrosinase inhibition was calculated using the formula: [(AC − AT)/AC] × 100. Next, mean values ± SD were calculated.

### 3.17. In Vitro Viability Assessment by the MTT Assay

Mouse melanoma B16F10 cells (American Type Culture Collection (ATCC, CRL-6475, Manassas, VA, USA) were cultured under standard conditions (37 °C, 5% CO_2_, 95% humidity) in Ham’s F-10 Nutrient Mix (Gibco, Waltham, MA, USA) supplemented with 10% fetal bovine serum (FBS, Gibco, Waltham, MA, USA) and antibiotics (1% streptomycin/penicillin mixture; Sigma–Aldrich, Darmstadt, Germany). The cells were seeded at the density of 2 × 10^3^ cells/mL on 96-well plates in Ham’s F-10 Nutrient Mix, 1% FBS, and 1% antibiotics. After 24 h, the cells were treated with the tested extracts or kojic acid and incubated for an additional 48 h. Next, MTT reagent (Sigma–Aldrich, Darmstadt, Germany) was added to each well and further incubated for 4 h. The formazan produced in the cells appeared as dark crystals at the bottom of the wells, which could be observed using an inverted microscope. Next, the medium was aspirated, and 100 µL of DMSO was added to each well. The absorbance was measured at 570 nm (A570) using a plate reader (Spectra iD3 Max, Molecular Devices; San Jose, CA, USA). Viability (% of control) was determined by dividing A570 of experimental wells (after incubation with the tested extracts) × 100% by A570 of control wells (after incubation with the solvent). Experiments were performed in triplicates [51,52].

### 3.18. In Vitro Melanin Production Assay

Mouse melanoma B16F10 cells (American Type Culture Collection (ATCC, CRL-6475, Manassas, VA, USA) were seeded at the density of 5 × 10^4^ cells/mL on 24-well plates and incubated in Dulbecco’s modified Eagle’s medium (DMEM, Gibco, Waltham, MA, USA) supplemented with 1% fetal bovine serum (FBS) (Gibco, Waltham, MA, USA) and antibiotics (1% streptomycin/penicillin mixture; Sigma–Aldrich, Darmstadt, Germany). After 24 h, the medium was replaced with new samples, containing either the tested extracts, a reference inhibitor, or a solvent with or without the addition of 1 µM α-MSH (Sigma–Aldrich, Darmstadt, Germany). The cells were further incubated under these conditions for 48 h. The cells were then washed twice with PBS and dissolved in 100 µL of 1M NaOH. Next, the plates were incubated at 60 °C for 1 h and mixed to solubilize melanin. The samples were then transferred to a 96-well plate, and the absorbance value was measured at 405 nm using a plate reader (Spectra iD3 Max, Molecular Devices, San Jose, CA, USA). The relative melanin content ± SD was found for 100%, and assigned to cells treated neither with extract nor kojic acid, with the absence of α-MSH [51].

### 3.19. Statistical Analysis

All experiments were performed in three replicates, with the results presented as mean ± standard deviation (SD). In addition, one-way analysis of variance (ANOVA) with Tukey’s post hoc test was conducted, and *p* < 0.05 was considered to be statistically significant. Statistical analysis was performed using XLSTAT 2016.

## 4. Conclusions

The study presents a comparative assessment of selected biological activities and mycochemical profiles of the biomass extracts of the medicinal fungi *Ganoderma* species. The selected species are of great interest because of their broad pharmacological potential. The results revealed that the biomass of the investigated species can be considered as an alternative to natural or cultivated material as a valuable raw material for the pharmaceutical or cosmetic industry, and its production can be controlled and stimulated.

## Figures and Tables

**Table 1:** Selected chemical compounds in the extract from biomass of *Ganoderma* spp. (mg/100 g d.w.).

Chemical Compounds	*Ganoderma adspersum*	*Ganoderma applanatum*	*Ganoderma carnosum*	*Ganoderma lucidum*	*Ganoderma pfeifferi*	*Ganoderma resinaceum*
Phenolic acids						
Gallic acid	17.62 ± 0.70	10.55 ± 1.44	nd	17.90 ± 2.57	34.31 ± 2.04	nd
Protocatechuic acid	3.07 ± 0.23	0.57 ± 0.39	3.95 ± 1.18	1.57 ± 0.07	1.86 ± 0.06	1.29 ± 0.99
3.4-Dihydroxyphenylacetic acid	38.95 ± 3.17	56.60 ± 2.73	nd	31.00 ± 1.50	77.37 ± 4.04	42.25 ± 11.52
*p*-Hydroxybenzoic acid	0.62 ± 0.15	1.83 ± 0.03	0.25 ± 0.08	0.09 ± 0.01	nd	2.17 ± 0.03
Caffeic acid	nd	0.60 ± 0.10	1.60 ± 0.07	nd	0.53 ± 0.06	nd
Non-hallucinogenic indoles						
L-tryptophan	6.02 ± 1.00	7.31 ± 1.02	3.03 ± 0.2	10.58 ± 2.07	7.26 ± 1.05	7.12 ± 1.06
Melatonin	nd	0.02 ± 0.004	nd	0.98 ± 0.02	0.012 ± 005	nd
Sterols						
Ergosterol	74.78 ± 1.27	28.15 ± 0.48	16.64 ± 0.69	28.72 ± 1.35	42.68 ± 2.30	44.26 ± 4.56
Ergosterol peroxide	*	*	*	*	*	*
Tyrosinase inhibitors						
Kojic acid	nd	0.14 ± 0.09	nd	0.39 ± 0.52	nd	nd

Values expressed are mean ± S.D. of three parallel measurements. nd: not detected; *: trace amount.

**Table 2:** Comparison of the total phenolic compounds, total flavonoid compounds, and antioxidant activity of biomass extracts using different methods *.

Assay	*Ganoderma adspersum*	*Ganoderma applanatum*	*Ganoderma carnosum*	*Ganoderma lucidum*	*Ganoderma pfeifferi*	*Ganoderma resinaceum*
TPC (mg GAE/g)	10.57 ± 0.09 ^d^	13.99 ± 0.08 ^b^	16.56 ± 0.37 ^a^	13.54 ± 0.37 ^b^	12.53 ± 0.11 ^c^	16.36 ± 0.38 ^a^
TFC (mg RE/g)	0.21 ± 0.02 ^c^	0.36 ± 0.03 ^b^	0.22 ± 0.02 ^c^	0.22 ± 0.01 ^c^	0.21 ± 0.04 ^c^	0.57 ± 0.02 ^a^
DPPH (mg TE/g)	5.61 ± 0.10 ^c^	5.83 ± 0.18 ^b^	5.01 ± 0.08 ^e^	5.27 ± 0.04 ^d^	5.28 ± 0.04 ^d^	6.74 ± 0.01 ^a^
ABTS (mg TE/g)	10.16 ± 0.02 ^b^	11.29 ± 0.28 ^a^	9.74 ± 0.03 ^bc^	9.61 ± 0.35 ^c^	9.77 ± 0.13 ^bc^	11.60 ± 0.36 ^a^
FRAP (mg TE/g)	7.27 ± 0.07 ^c^	8.10 ± 0.05 ^b^	7.40 ± 0.22 ^c^	6.91 ± 0.05 ^d^	8.10 ± 0.13 ^b^	10.16 ± 0.08 ^a^
CUPRAC (mg TE/g)	17.50 ± 0.45 ^e^	23.55 ± 0.24 ^c^	22.94 ± 0.27 ^c^	20.35 ± 0.84 ^d^	25.28 ± 0.24 ^b^	31.78 ± 0.33 ^a^
PHD (mmol TE/g)	0.45 ± 0.02 ^e^	0.52 ± 0.02 ^cd^	0.53 ± 0.01 ^bc^	0.57 ± 0.03 ^ab^	0.48 ± 0.01 ^de^	0.61 ± 0.04 ^a^
MCA (mg EDTAE/g)	8.51 ± 0.09 ^a^	8.98 ± 0.10 ^a^	8.49 ± 0.78 ^a^	7.74 ± 0.96 ^ab^	5.20 ± 0.52 ^c^	6.40 ± 0.79 ^bc^

* Values expressed are mean ± S.D. of three parallel measurements. TPC: total phenolic content; TFC: total flavonoid content; ABTS: 2,2′-azino-bis(3-ethylbenzothiazoline) 6-sulfonic acid; DPPH: 1,1-diphenyl-2-picrylhydrazyl; CUPRAC: cupric ion reducing antioxidant capacity; FRAP: ferric ion reducing antioxidant power; PHD: phosphomolybdenum; MCA: metal chelating activity; TE: trolox equivalent; EDTAE: EDTA equivalent. GAE: gallic acid equivalent; and RE: rutin equivalent. Different letters on the same rows indicate the differences in the tested samples (*p* < 0.05, from ANOVA, Tukey’s post hoc test).

**Table 3:** Comparison of the inhibition of selected enzymes by biomass extracts *.

Assay	*Ganoderma adspersum*	*Ganoderma applanatum*	*Ganoderma carnosum*	*Ganoderma lucidum*	*Ganoderma pfeifferi*	*Ganoderma resinaceum*
AChE (mg GALAE/g)	1.22 ± 0.01 ^a^	na	1.19 ± 0.01 ^c^	na	1.20 ± 0.01 ^b^	na
BChE (mg GALAE/g)	1.09 ± 0.08 ^a^	1.13 ± 0.13 ^a^	0.70 ± 0.02 ^c^	0.90 ± 0.03 ^b^	0.60 ± 0.03 ^c^	0.86 ± 0.08 ^b^
Amylase (mmol ACAE/g)	0.15 ± 0.01 ^a^	0.14 ± 0.01 ^bc^	0.14 ± 0.01 ^c^	0.14 ± 0.01 ^b^	0.14 ± 0.01 ^c^	0.13 ± 0.01 ^d^

* Values are expressed are mean ± S.D. of three parallel measurements. AChE: acetylcholinesterase; BChE: butrylcholinesterase; GALAE: galantamine equivalent; KAE: kojic acid equivalent; ACAE: acarbose equivalent; na: not active. Different letters on the same lines indicate the differences in the tested samples (*p* < 0.05, from ANOVA, Tukey’s post hoc test).

**Table 4:** Tyrosinase inhibition properties of the tested extracts at the concentration of 5 mg/mL or kojic acid at the concentration of 0.1 mg/mL with 0.5 mM L–DOPA.

Tested Extract or Reference	*Ganoderma adspersum*	*Ganoderma applanatum*	*Ganoderma carnosum*	*Ganoderma lucidum*	*Ganoderma pfeifferi*	*Ganoderma resinaceum*	Kojic Acid
% of Inhibition ± SD	0	0	0	50.53 ± 3.23	29.18 ± 0.44	45.31 ± 0.87	81.59 ± 0.61

## Data Availability

The data are contained within this article.

## Sample Availability

Samples of the compounds are available from the authors.

## References

[1] Karsten P. (1881). Enumeratio Boletinarum et Polyporarum Fennicarum systemate novo dispositorum. Rev. Mycol..

[2] Wang L., Li J.Q., Zhang J., Li Z.M., Liu H.G., Wang Y.Z. (2020). Traditional uses, chemical components and pharmacological activities of the genus *Ganoderma* P. Karst.: A review. RSC Adv..

[3] Basnet B.B., Liu L., Bao L., Liu H. (2017). Current and future perspective on antimicrobial and anti-parasitic activities of *Ganoderma* sp.: An update. Mycology.

[4] Jonathan S.G., Awotona F.E. (2010). Studies on antimicrobial potentials of three *Ganoderma* species. Afr. J. Biomed. Res..

[5] Xia Q., Zhang H., Sun X., Zhao H., Wu L., Zhu D., Yang G., Shao Y., Zhang X., Mao X. (2014). A comprehensive review of the structure elucidation and biological activity of triterpenoids from *Ganoderma* spp.. Molecules.

[6] Shiao M.S. (2003). Natural products of the medicinal fungus *Ganoderma lucidum*: Occurrence, biological activities, and pharmacological functions. Chem. Rec..

[7] Ansor N.M., Abdullah N., Aminudin N. (2013). Anti-angiotensin converting enzyme (ACE) proteins from mycelia of *Ganoderma lucidum* (Curtis) P. Karst. BMC Complement Altern. Med..

[8] Hirotani M., Ino C., Furuya T., Shiro M. (1986). Ganoderic acids T, S and R, new triterpenoids from the cultured mycelia of *Ganoderma lucidum*. Chem. Pharm. Bull..

[9] Gibbs P.A., Seviour R.J., Schmid F. (2000). Growth of filamentous fungi in submerged culture: Problems and possible solutions. Crit. Rev. Biotechnol..

[10] Mooralitharan S., Hanafiah Z.M., Manan T.S.B.A., Hasan H.A., Jensen H.S., Wan-Mohtar W.A.A.Q.I., Mohtar W.H.M.W. (2021). Optimization of mycoremediation treatment for the chemical oxygen demand (COD) and ammonia nitrogen (AN) removal from domestic effluent using wild-Serbian *Ganoderma lucidum* (WSGL). Environ. Sci. Pollut. Res..

[11] Yue G.G.L., Fung K.P., Tse G.M.K., Leung P.C., Lau C.B.S. (2006). Comparative studies of various *Ganoderma* species and their different parts with regard to their antitumor and immunomodulating activities in vitro. J. Altern. Complement. Med..

[12] Sułkowska-Ziaja K., Szewczyk A., Galanty A., Gdula-Argasińska J., Muszyńska B. (2018). Chemical composition and biological activity of extracts from fruiting bodies and mycelial cultures of *Fomitopsis betulina*. Mol. Biol. Rep..

[13] Fijałkowska A., Muszyńska B., Sułkowska-Ziaja K., Kała K., Pawlik A., Stefaniuk D., Matuszewska A., Piska K., Pękala E., Kaczmarczyk P. (2020). Medicinal potential of mycelium and fruiting bodies of an arboreal mushroom *Fomitopsis officinalis* in therapy of lifestyle diseases. Sci. Rep..

[14] Heleno S.A., Martins S., Queiroz M.J.R.P., Ferreira I.C.F.R. (2015). Bioactivity of phenolic acids: Metabolites versus parent compounds: A review. Food Chem..

[15] Khadem S., Marles R.J. (2010). Monocyclic phenolic acids; hydroxy– and polyhydroxybenzoic acids: Occurrence and recent bioactivity studies. Molecules.

[16] Karaman M., Jovin E., Malbaša R., Matavuly M., Popović M. (2010). Medicinal and edible lignicolous fungi as natural sources of antioxidative and antibacterial agents. Phytother. Res..

[17] Rašeta M., Popović M., Beara I., Šibul F., Zengin G., Krstić S., Karaman M. (2021). Anti-inflammatory, antioxidant and enzyme inhibition activities in correlation with mycochemical profile of selected indigenous *Ganoderma* spp. from Balkan Region (Serbia). Chem. Biodivers..

[18] Wang S.Y., Shi X.C., Laborda P. (2020). Indole-based melatonin analogues: Synthetic approaches and biological activity. Review. Eur. J. Med. Chem..

[19] Muszyńska B., Sułkowska-Ziaja K. (2015). Impact of food processing on non-hallucinogenic indole derivatives in edible mushrooms. Processing and Impact on Active Components in Food.

[20] Sułkowska-Ziaja K., Maślanka A., Szewczyk A., Muszyńska B. (2017). Physiologically active compounds in four species of *Phellinus*. Nat. Prod. Commun..

[21] Barreira J.C.M., Oliveira M.B.P.P., Ferreira I.C.F.R. (2014). Development of a Novel Methodology for the Analysis of Ergosterol in Mushrooms. Food Anal. Methods.

[22] Rosecke J., Konig W.A. (2000). Constituents of various wood-rotting basidiomycetes. Phytochemistry.

[23] Lv G.P., Zhao J., Duan J.A., Tang Y.P., Li S.P. (2012). Comparison of sterols and fatty acids in two species of *Ganoderma*. Chem. Cent. J..

[24] Kiss A., Grünvald P., Ladányi M., Papp V., Papp I., Némedi E., Mirmazloum I. (2021). Heat treatment of Reishi medicinal mushroom (*Ganoderma lingzhi*) basidiocarp enhanced its β-glucan solubility, antioxidant capacity and lactogenic properties. Foods.

[25] Tan E.S.S., Leo T.K., Tan C.K. (2021). Effect of tiger milk mushroom (*Lignosus rhinocerus*) supplementation on respiratory health, immunity and antioxidant status: An open-label prospective study. Sci. Rep..

[26] Zengin G., Sarikurkcu C., Gunes E., Uysal A., Ceylan R., Uysal S., Gungor H., Aktumsek A. (2015). Two *Ganoderma* species: Profiling of phenolic compounds by HPLC–DAD, antioxidant, antimicrobial and inhibitory activities on key enzymes linked to diabetes mellitus, Alzheimer’s disease and skin disorders. Food Funct..

[27] Saltarelli R., Palma F., Gioacchini A.M., Calcabrini C., Mancini U., De Bellis R., Stocchi V., Potenza L. (2019). Phytochemical composition, antioxidant and antiproliferative activities and effects on nuclear DNA of ethanolic extract from an Italian mycelial isolate of *Ganoderma lucidum*. J. Ethnopharmacol..

[28] Sharpe E., Farragher-Gnadt A.P., Igbanugo M., Huber T., Michelotti J.C., Milenkowic A., Ludlam S., Walker M., Hanes D., Bradley R. (2021). Comparison of antioxidant activity and extraction techniques for commercially and laboratory prepared extracts from six mushroom species. J. Sci. Food Agric..

[29] Cho J.Y., Sadiq N.B., Kim J.C., Lee B., Hamayun M., Lee T.S., Kim H.Y. (2021). Optimization of antioxidant, anti-diabetic, and anti-inflammatory activities and ganoderic acid content of differentially dried *Ganoderma lucidum* using response surface methodology. Food Chem..

[30] Heleno S.A., Barros L., Martins A., Queiroz M.J.R., Santos-Buelga C., Ferreira I.C.F.R. (2012). Fruiting body, spores and in vitro produced mycelium of *Ganoderma lucidum* from Northeast Portugal: A comparative study of the antioxidant potential of phenolic and polysaccharidic extracts. Food Res. Int..

[31] Tel-Çayan G., Öztürk M., Duru M.E., Rehman M.U., Adhikari A., Türkoğlu A., Choudhary M.I. (2015). Phytochemical investigation, antioxidant and anticholinesterase activities of *Ganoderma adspersum*. Ind Crops Prod..

[32] Taofiq O., Heleno S.A., Calhelha R.C., Alves M.J., Barros L., González-Paramás A.M., Barreiro M.F., Ferreira I.C.F.R. (2017). The potential of *Ganoderma lucidum* extracts as bioactive ingredients in topical formulations, beyond its nutritional benefits. Food Chem. Toxicol..

[33] Saltarelli R., Ceccaroli P., Buffalini M., Vallorani L., Casadei L., Zambonelli A., Stocchi V., Iotti M., Badalyan S., Stocchi V. (2015). Biochemical characterization and antioxidant and antiproliferative activities of different *Ganoderma collections*. J. Mol. Microbiol..

[34] Sharma C., Bhardwaj N., Sharma A., Tuli H.S., Batra P., Beniwal V., Gupta G.K., Sharma A.K. (2019). Bioactive metabolites of *Ganoderma lucidum*: Factors, mechanism and broad spectrum therapeutic potential. J. Herb. Med..

[35] Wei J.C., Wang A.H., Wei Y.L., Huo X.K., Tian X.G., Feng L., Ma X.C., Wang C., Huang S.S., Jia J.M. (2018). Chemical characteristics of the fungus *Ganoderma lucidum* and their inhibitory effects on acetylcholinesterase. J. Asian Nat. Prod. Res..

[36] Kaur A., Randhawa K., Singh V., Shri R. (2019). Bioactivity-guided isolation of acetylcholinesterase inhibitor from *Ganoderma mediosinense* (Agaricomycetes). Int. J. Med. Mushrooms.

[37] Deveci E., Çayan F., Tel-Çayan G., Duru M.E. (2021). Inhibitory activities of medicinal mushrooms on α-amylase and α-glucosidase-enzymes related to type 2 diabetes. S. Afr. J. Bot..

[38] Yalcin O.U., Sarikurkcu C., Cengiz M., Gungor H., Zeljković S.C. (2020). *Ganoderma carnosum* and *Ganoderma pfeifferi:* Metal concentration, phenolic content, and biological activity. Mycologia.

[39] Chen S.D., Yong T.Q., Zhang Y.F., Hu H.P., Xie Y.Z. (2019). Inhibitory Effect of Five *Ganoderma* Species (Agaricomycetes) against Key Digestive Enzymes Related to Type 2 Diabetes Mellitus. Int. J. Med. Mushrooms.

[40] Subramaniam S., Raman J., Sabaratnam V., Heng C.K., Kuppusamy U.R. (2017). Functional properties of partially characterized polysaccharide from the medicinal mushroom *Ganoderma neo-japonicum* (Agaricomycetes). Int. J. Med. Mushrooms.

[41] Long Z., Xue Y., Ning Z., Sun J., Li J., Su Z., Liu Q., Xu C., Yan J.K. (2021). Production, characterization, and bioactivities of exopolysaccharides from the submerged culture of *Ganoderma cantharelloideum* MH Liu. 3 Biotech.

[42] Joo O.S., Hwang C.E., Hong S.Y., Sin E.C., Nam S.H., Cho K.M. (2018). Antioxidative and digestion enzyme inhibitory activity of *Ganoderma lucidum* depends on the extraction solvent. Korean J. Food Preserv..

[43] Migas P., Borys I. (2021). BMD–TLC—A useful separation technique for quantitative analysis of arbutin and hydroquinone in herbal drugs. Acta Chromatogr..

[44] Noh J.M., Kwak S.Y., Seo H.S., Seo J.H., Kim B.G., Lee Y.S. (2009). Kojic acid–amino acid conjugates as tyrosinase inhibitors. Bioorganic Med. Chem. Lett..

[45] Oddoux L. (1957). Recherches sur les Myceliums Secondaires des Homobasidie’s en Culture; Morphologie, Cytologie, Exigences Alimentaires.

[46] Włodarczyk A., Krakowska A., Sułkowska-Ziaja K., Suchanek M., Zięba P., Opoka W., Muszyńska B. (2021). *Pleurotus* spp. Mycelia enriched in magnesium and zinc Salts as a potential functional food. Molecules.

[47] Zengin G., Aktumsek A. (2014). Investigation of antioxidant potentials of solvent extracts from different anatomical parts of *Asphodeline anatolica* E. Tuzlaci: An endemic plant to Turkey. Afr. J. Tradit. Complement. Altern. Med..

[48] Zengin G., Nithiyanantham S., Locatelli M., Ceylan R., Uysal S., Aktumsek A., Selvi P.K., Maskovic P. (2016). Screening of in vitro antioxidant and enzyme inhibitory activities of different extracts from two uninvestigated wild plants: *Centranthus longiflorus* subsp. longiflorus and Cerinthe minor subsp. auriculata. Eur. J. Integr. Med..

[49] Zengin G. (2016). A study on in vitro enzyme inhibitory properties of *Asphodeline anatolica*: New sources of natural inhibitors for public health problems. Ind. Crops. Prod..

[50] Saghaie L., Pourfarzam M., Fassihi A., Sartippour B. (2013). Synthesis and tyrosinase inhibitory properties of some novel derivatives of kojic acid. Res. Pharm. Sci..

[51] Bae S.J., Ha Y.M., Park Y.J., Song Y.M., Ha T.K., Chun P., Moon H.R., Chung H.Y. (2012). Design, synthesis, and evaluation of (E)-N-substituted benzylidene-aniline derivatives as tyrosinase inhibitors. Eur. J. Med. Chem..

[52] Popiół J., Gunia-Krzyżak A., Słoczyńska K., Koczurkiewicz P., Piska K., Wójcik-Pszczoła K., Żelaszczyk D., Krupa A., Żmudzki P., Marona H. (2021). The involvement of xanthone and (E)-cinnamoyl chromophores for the design and synthesis of novel sunscreening agents. Int. J. Mol..

